# Microvascular obstruction in cardiac amyloidosis

**DOI:** 10.1002/ejhf.3481

**Published:** 2024-10-18

**Authors:** Lucrezia Netti, Adam Ioannou, Ana Martinez‐Naharro, Yousuf Razvi, Aldostefano Porcari, Lucia Venneri, Viviana Maestrini, Dan Knight, Ruta Virsinskaite, Muhammad U. Rauf, Tushar Kotecha, Rishi K. Patel, Ashutosh Wechelakar, Helen Lachmann, Peter Kellman, Charlotte Manisty, James Moon, Philip N. Hawkins, Julian D. Gillmore, Marianna Fontana

**Affiliations:** ^1^ Department of Clinical, Internal, Anesthesiological and Cardiovascular Sciences Sapienza University of Rome Rome Italy; ^2^ National Amyloidosis Centre University College London, Royal Free Campus London UK; ^3^ Center for Diagnosis and Treatment of Cardiomyopathies, Cardiovascular Department Azienda Sanitaria Universitaria Giuliano‐Isontina (ASUGI), University of Trieste Trieste Italy; ^4^ National Heart, Lung and Blood Institute, National Institutes of Health Bethesda MD USA; ^5^ St Bartholomew's Hospital London UK

## Introduction

Cardiac amyloidosis (CA) is caused by deposition of misfolded proteins in the form of amyloid fibrils within the myocardial extracellular space, which disrupt myocardial structure and function, but also infiltrate the vascular wall and disrupt the capillary architecture.^1^, ^2^ Cardiac magnetic resonance (CMR) with multiparametric mapping enables detailed tissue characterization, and quantitative perfusion imaging has demonstrated severe inducible ischaemia in patients with CA, in the absence of significant epicardial coronary artery disease.^3^, ^4^ Microvascular obstruction (MVO) is a marker of injury of the microvasculature related to a no‐reflow phenomenon, where blood flow cannot penetrate beyond the vascular bed. MVO can be characterized with CMR and is associated with a worse prognosis following an acute myocardial infarction.^5^

The aims of this study are to characterize the prevalence of MVO in patients with CA and assess the association between MVO and prognosis.

## Methods

This retrospective observational cohort comprised individuals with light‐chain CA (AL‐CA) and transthyretin CA (ATTR‐CA) who underwent a CMR including early gadolinium enhancement images at diagnosis at the National Amyloidosis Centre (2016–2021). Patients with known coronary artery disease were excluded (online supplementary *Figure* *S1*). Patients were managed in accordance with the Declaration of Helsinki and provided written informed consent for analysis and publication of their data. Blood biomarker testing and cardiac imaging protocols are described in the online supplementary *Appendix*.

Continuous variables are presented as mean ± standard deviation or median with interquartile range. Continuous variables were analysed using the independent *t*‐test or one‐way analysis of variance if data were normally distributed; or their non‐parametric equivalents if data were not normally distributed. Categorical data are presented as absolute numbers and frequencies (%) and compared using the *χ* ^2^ test. Univariable and multivariable binary logistic regression analysis was used to investigate parameters associated with MVO. Each variable was tested at binary logistic regression analysis for the presence of MVO and those significant at *p* < 0.1 were included in a multivariable binary logistic regression analysis.

All mortality data were obtained via the UK Office of National Statistics, which is the national government registry for all deaths. Survival was evaluated with Cox proportional hazards regression analysis. Kaplan–Meier curves were constructed, with statistical significance being assessed with a log‐rank test. Statistical significance was defined as *p* < 0.05.

## Results

The study included 800 patients (400 AL‐CA and 400 ATTR‐CA patients), with a median age 73 years (65–79), and 608 (76%) were male. Baseline characteristics of the study population are shown in *Table* *1*. A total of 221 (27.6%) patients were found to have MVO at diagnosis, 97 (43.9%) of whom had AL‐CA and 124 (56.1%) ATTR‐CA. Patients with MVO on CMR had a more severe cardiac phenotype as evidenced by high serum cardiac biomarkers, worse echocardiographic and CMR derived parameters of cardiac structure and function, and a higher amyloid load as evidenced by a greater burden of late gadolinium enhancement and higher ECV, than those without MVO (*Table* *1*). The differences in cardiac structure, function and tissue characterization between patients with and without MVO remained significant in the subgroups of patients with AL‐CA and ATTR‐CA (online supplementary *Tables* *S1* and *S2*). Multivariable logistic regression demonstrated that after adjusting for demographic, biomarker, structural and functional parameters, extracellular volume (ECV) had the strongest association with MVO (odds ratio 1.05, 95% CI 1.02–1.08, *p* < 0.001) (online supplementary *Table* *S3*).

**Table 1:** Baseline characteristics of the study population stratified by absence or presence of microvascular obstruction

Characteristics	All (*n* = 800)	No MVO (*n* = 579, 72%)	MVO (*n* = 221, 28%)	*p*‐value
Male sex	76% (608)	73% (421)	85% (187)	**<0.001**
ATTR‐CA	50% (400)	48% (276)	56% (124)	**0.033**
Age at baseline (years)	73 (65–79)	73 (64–79)	72 (67–83)	0.936
Serum biomarkers				
NT‐proBNP (ng/ml)	2863 (1326–5856)	2508 (1203–5752)	3516 (1944–6247)	**<0.001**
Troponin (ng/ml)	61 (37–97)	59.5 (35–95)	67 (45–103)	**0.003**
eGFR (ml/min/1.73 m^2^)	65 (52–83)	66 (53–85)	63 (51–79)	0.257
Echocardiographic parameters				
GLS (%)	−11.5 (−9; −15)	−12 (−9.2; −16)	−10.5 (−7.9; −12.6)	**<0.001**
E/e'	15.4 (11.3–19.9)	15 (11–19.2)	16.4 (12.8–21)	**0.001**
CMR parameters				
LVEDV_i_ (ml/m^2^)	65 (55–76)	64 (54–75)	66 (58–79)	**0.005**
LVESV_i_ (ml/m^2^)	25 (18–34)	24 (17–33)	29 (22–38)	**<0.001**
MWT (mm)	17 (15–20)	17 (14–20)	19 (16–21)	**<0.001**
LV mass_i_ (g/m^2^)	112 (89–137)	107 (84–130)	125 (103.5–147.5)	**<0.001**
LAA_i_ (cm^2^/m^2^)	16 (14–18)	16 (13–18)	16.5 (14–19)	**0.002**
EF (%)	60 (50–69)	62 (51–70)	57 (48–64)	**<0.001**
SV_i_ (ml/m^2^)	38 (31–45)	38 (32–46)	37 (31–44)	0.214
TAPSE (mm)	13 (9–18)	14 (9–19)	11 (8–15)	**<0.001**
T1 (ms)	1157 (1122–1196)	1154 (1119–1193)	1166 (1137–1203)	**0.001**
T2 (ms)	51 (49–54)	51 (49–53)	51 (49–54)	0.364
Transmural LGE	67.5% (539)	61% (353)	84.5% (186)	**<0.001**
RV LGE	82.6% (660)	78.8% (456)	92.7% (204)	**<0.001**
ECV (%)	52.5 (46–58)	50 (45–57)	56 (51–61)	**<0.001**

ATTR‐CA, transthyretin cardiac amyloidosis; CMR, cardiac magnetic resonance; ECV, extracellular volume; EF, ejection fraction; eGFR, estimated glomerular filtration rate; LAA_i_, indexed left atrial area; GLS, global longitudinal strain; LGE, late gadolinium enhancement; LV, left ventricle; LVEDV_i_, indexed left ventricular end‐diastolic volume; LVESV_i_, indexed left ventricular end‐systolic volume; MVO, microvascular obstruction; MWT, maximal wall thickness; NT‐proBNP, N‐terminal pro‐brain natriuretic peptide; RV, right ventricle; SV_i_, indexed stroke volume; TAPSE, tricuspid annular plane systolic excursion.

**Figure 1:**
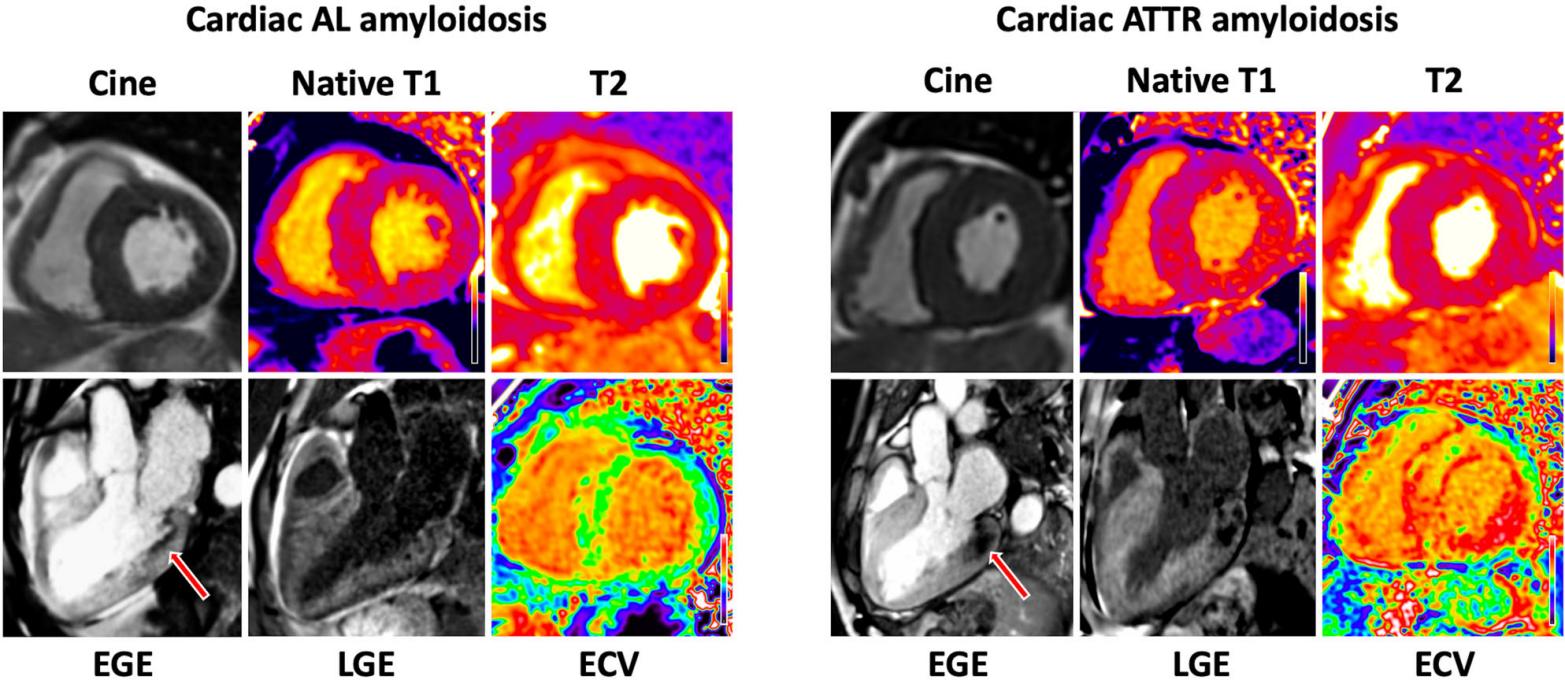
(Left panel) Cardiac magnetic resonance imaging in cardiac light‐chain (AL) amyloidosis demonstrating subendocardial microvascular obstruction (arrow). (Right panel) Cardiac magnetic resonance imaging in cardiac transthyretin amyloidosis (ATTR) demonstrating subendocardial‐to‐midwall microvascular obstruction (arrow). ECV, extracellular volume; EGE, early gadolinium enhancement; LGE, late gadolinium enhancement.

Patients with AL‐CA and MVO had higher N‐terminal pro‐brain natriuretic peptide (5184 ng/L [2602–9835] vs. 2725 ng/L [1291–4876], *p* < 0.001) and troponin T (86 ng/L [47–148] vs. 59 ng/L [44–78], *p* < 0.001), whereas patients with ATTR‐CA and MVO had larger left ventricular volumes (indexed left ventricular end‐diastolic volume 71 ml/m^2^ [60–84] vs. 62 ml/m^2^ [55–74], *p* = 0.001); (indexed left ventricular end‐systolic volume 31.5 ml/m^2^ [23.3–39.8] vs. 26 ml/m^2^ [20–34], *p* = 0.002) and a greater indexed left ventricular mass (138 g/m^2^ [115–159] vs. 113 g/m^2^ [96–137], *p* < 0.001). Patients with AL‐CA and MVO had a higher native T1 (1188 ms [1157–1230] vs. 1151 ms [1121–1186], *p* < 0.001) and T2 (53 ms [50–56] vs. 50 ms [48–52], *p* < 0.001), but lower ECV (55% [50–60] vs. 58% [53–61], *p* = 0.008) and lower indexed myocyte cell volume (48.6 g/m^2^ [41.1–59.8] vs. 55.7 g/m^2^ [47.5–68.4], *p* < 0.001) than patients with ATTR‐CA and MVO. LGE was more commonly transmural (117 [94.3%] vs. 70 [72.2%], *p* < 0.001) and involved both ventricles in patients with ATTR‐CA and MVO than patients with AL‐CA and MVO (120 [96.7%] vs. 85 [87.6%], *p* < 0.001) (*Figure* *1* and online supplementary *Table* *S4*).

Both patients with AL‐CA and ATTR‐CA had a similar distribution of MVO, which was most often present in the basal segments (AL: 94 [96.9%], ATTR: 118 [95.6%]), and MVO in the basal segments was most commonly found in the basal lateral segment (AL: 82 [87.2%], ATTR: 107 [90.7%]). The majority of MVO was confined to the subendocardium (*n* = 97, 75.3%), however in patients with ATTR‐CA a significantly higher proportion demonstrated extension of the MVO from the subendocardium into the mid‐wall, compared with AL‐CA (50 [40.3%] vs. 15 [15.5%], *p* < 0.001).

**Figure 2:**
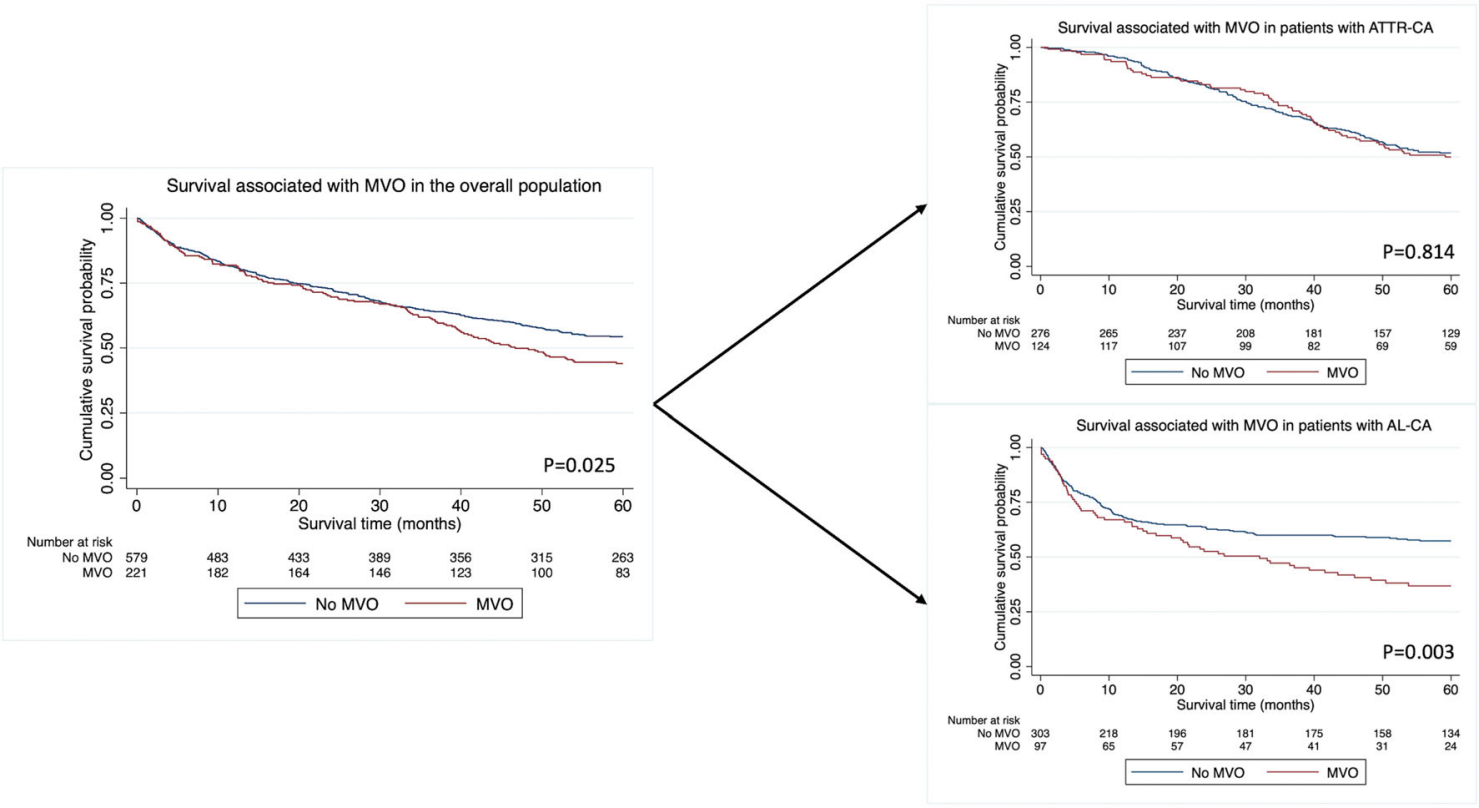
Kaplan–Meier survival curves displaying survival according to either the presence or the absence of microvascular obstruction (MVO) in all patients (left panel), in patients with transthyretin cardiac amyloidosis (ATTR‐CA) (right, top panel) and in patients with light‐chain cardiac amyloidosis (AL‐CA) (right, bottom panel).

At a median follow up of 51.5 months (19–60), 381 patients died and the death rate was 14.1 deaths per 100 person‐years (py) (95% confidence interval [CI] 12.7–15.6). In patients with MVO the death rate was 16.9 deaths per 100py (95% CI 14.1–20.1) compared with 13.1 deaths per 100py (95% CI 11.5–14.8) in patients without MVO. In the overall population MVO was associated with a 28% increased risk of death (hazard ratio [HR] 1.28, 95% CI 1.03–1.59, *p* = 0.025). Subgroup analysis demonstrated that MVO was associated with a 60% increased risk of death in AL‐CA (HR 1.59, 95% CI 1.17–2.17, *p* = 0.003) and remained independently associated with mortality after adjusting for the Mayo stage (HR 1.39, 95% CI 1.02–1.89, *p* = 0.039), but there was no convincing evidence for a difference in mortality in patients with ATTR‐CA (HR 1.04, 95% CI 0.77–1.40, *p* = 0.814) (*Figure* *2*).

## Discussion

This study demonstrates that MVO is present in just over one‐quarter of patients with CA, and is more common in ATTR‐CA than AL‐CA, and associated with a more severe cardiac phenotype. MVO is associated with an increased risk of mortality in patients with AL‐CA, but not in ATTR‐CA.

The exact pathophysiological mechanisms responsible for MVO in CA remain elusive. Multiple autopsy studies of explanted hearts have demonstrated obstruction of intramural vessels secondary to amyloid infiltration, while endomyocardial biopsies have demonstrated distortion of microvascular architecture and rarefaction of the capillaries, alongside obstruction of intramural vessels. Subendocardial and interstitial myocardial fibrosis is only present in a minority of cases and is mostly mild, whereas morphological myocyte alterations that represent chronic damage in a remodelled myocardium consequent to amyloid deposits, such as myocyte attenuation/atrophy, myocyte vacuolization and focal reactive myocyte hypertrophy, are extremely common, suggesting the changes are secondary to direct amyloid infiltration. The histological changes in the vasculature and cardiomyocytes, combined with up‐regulation of vascular endothelial growth factor strongly suggest myocardial ischaemia, and this has been confirmed by studies that used stress perfusion imaging to demonstrate that patients with CA have a profound reduction in stress myocardial blood flow and myocardial perfusion reserve.^3^, ^6^

The burden of MVO correlates with the magnitude of amyloid infiltration, as measured by ECV, and also with the severity of cardiac phenotype as evidenced by a wide array of serum biomarkers, and structural and functional cardiac imaging parameters. Patients with ATTR‐CA had a greater prevalence and burden of MVO than patients with AL‐CA, which is likely to reflect that patients with ATTR‐CA often have a more advanced cardiac phenotype at diagnosis.

Despite the higher prevalence of MVO in patients with ATTR‐CA, MVO was only associated with an increased risk of mortality in patients with AL‐CA. This disparity may be explained by inherent differences in disease biology between ATTR and AL‐CA. In the context of AL‐CA, in addition to amyloid fibril deposition within the extracellular matrix, amyloidogenic light chains exert a cardiotoxic effect on cardiomyocytes through proteome remodelling, activation of oxidative stress pathways and induction of apoptosis.^7^ It is possible that due to light‐chain toxicity, patients with AL‐CA are more susceptible to MVO, which in turn accelerates myocyte death, and this could explain the higher serum troponin T and myocardial T2 (a marker of myocardial oedema), and lower myocyte cell volume observed in patients with AL‐CA and MVO, despite patients with ATTR‐CA and MVO having a higher ECV.

This observation may have important implications in the development of novel therapies designed to remove amyloid fibrils from the heart, and the presence of MVO may impair delivery and the degree of target engagement. It is also possible that removal of amyloid fibrils may enable resolution of MVO, which in turn may facilitate cardiomyocyte recovery and represent a marker of treatment response.^8^, ^9^

## Conclusions

In summary, MVO is common in CA and is related to the magnitude of amyloid infiltration. MVO is associated with an increased risk of mortality in AL‐CA, but not in ATTR‐CA, highlighting intrinsic differences in disease biology between these two forms of CA. However, this is a single‐centre study, and therefore these results require external validation.

### Funding

Marianna Fontana is supported by a British Heart Foundation Intermediate Clinical Research Fellowship (FS/18/21/33447). Daniel Knight is supported by a British Heart Foundation Clinical Research Leave Fellowship (FS/CRLF/20/23004).

**Conflict of interest**: A.W. has consulting income from Alexia, AstraZeneca, Janssen, Attralus and Prothena. P.H. has consulting income from Alnylam. J.D.G. has consulting income from Ionis, Alexion, Eidos, Intellia, Alnylam and Pfizer. M.F. has consulting income from Intellia, Novo‐Nordisk, Pfizer, Eidos, Prothena, Alnylam, Alexion, Janssen and Ionis. All other authors have nothing to disclose.

## Supporting information


**Appendix S1.** Supporting Information.
