# Supplementary material for: Microvascular obstruction in cardiac amyloidosis
Source: Eur J Heart Fail. 2024 Oct 18;27(12):2948–51. doi: 10.1002/ejhf.3481 (PMC12803619; doi:10.1002/ejhf.3481)
Supplement: Supplementary file 1 — Appendix S1. Supporting Information. [file EJHF-27-2948-s001.docx]

**Appendix**

**Biomarkers**

N-Terminal pro-B-type natriuretic peptide was measured with an electrochemiluminescence sandwich immunoassay on the Elecsys system 2010 (Roche Diagnostics). Glomerular filtration rate was estimated (eGFR) according to the standard Modification of Diet in Renal Disease (MDRD) formula (including the correction for race). High-sensitivity troponin T assay was performed with a second-generation assay.

**Echocardiographic image acquisition and analysis**

Echocardiographic evaluation was performed using a GE Vivid E9 ultrasound machine equipped with a 5S probe; at least three consecutive beats were recorded for each view, images were stored and analysis and measurements were performed offline using the most up-to-date EchoPAC software at the time of image acquisition according to current recommendations. Among the echocardiographic parameters we considered the LV diastolic filling pressures and the global longitudinal strain (GLS). LV early diastolic filling (E wave) was evaluated with pulsed Doppler in the 4-chamber view. Lateral mitral annulus velocity (lateral e′ wave) was assessed with tissue Doppler in the 4-chamber view; the ratio between the LV early diastolic filling wave and lateral mitral annulus velocity (E/e′) was calculated. Clips of the apical 4-chamber, 2-chamber and 3-chamber views with at least three cardiac cycles, high frame rates (70 to 100 frame/s) and without dropout of LV segments or significant foreshortening of the ventricle were considered suitable for offline 2D speckle strain imaging analysis. The endocardial border was traced at the end-diastolic frame in all these views. The software tracked speckles along the endocardial and epicardial borders throughout the cardiac cycle, and the width of the region of interest was modified to fit the entire myocardium. Peak longitudinal strain was computed automatically, generating regional data from 17 segments, to calculate an average value. All echocardiographic image analysis was performed blinded to all other clinical and imaging data.

**CMR acquisition and analysis**

All participants underwent CMR on a 1.5-T scanner (Magnetom Aera, Siemens Healthcare, Erlangen, Germany). Within a conventional clinical scan (localizers and cine imaging with steady state free precession (SSFP) sequence), early gadolinium enhancement (EGE) and late gadolinium enhancement (LGE) imaging were acquired after a bolus of gadoterate meglumine (0.1 mmol/kg, gadolinium-DOTA, Dotarem, Guerbet S.A. France). Images using magnitude inversion recovery (MAGIR) and phase-sensitive inversion recovery (PSIR) sequence reconstructions with SSFP read-outs were acquired after from immediately to 14 minutes after gadolinium contrast administration using a motion corrected protocol. Long axis and short axis images were acquired to optimize for early and late image acquisition. Inversion time was typically set up at 230 ms and increased progressively. For native T1 mapping, basal and midventricular short axis, and 4-chamber long-axis were acquired using the modified LookLocker inversion recovery (MOLLI) sequence after regional shimming. Post-contrast T1 mapping (post-MOLLI) was performed using the same sequence and slice positions 15 minutes post-contrast. T1-mapping protocols used 5s(3s)3s and 4s(1s)3s(1s)2s sampling, pre- and post-contrast, respectively. For T2 mapping, a 4-chamber long-axis matching the T1 map was acquired using an investigational prototype (WIP 448B, Siemens Healthcare, Erlangen, Germany). This sequence uses 3 single-shot T2-prepared steady state free precession (SSFP) readouts each separated with 3 heartbeats for T1 recovery. The echo times (TE) for the individual T2 preparations were 0.25, and 45 ms.

All CMR image analysis was performed blinded to all other clinical and imaging data. EGE and LGE images were retrospectively re-assessed to qualitatively describe the presence of microvascular obstruction (MVO), as a dark area within the myocardium that does not enhance few minutes after gadolinium administration and that enhances after on LGE images. The location of MVO throughout the left ventricle was described in accordance with the American Heart Association 16-segment model and the distribution was described as being either subendocardial or mid-wall.

**Supplementary Figure S1**


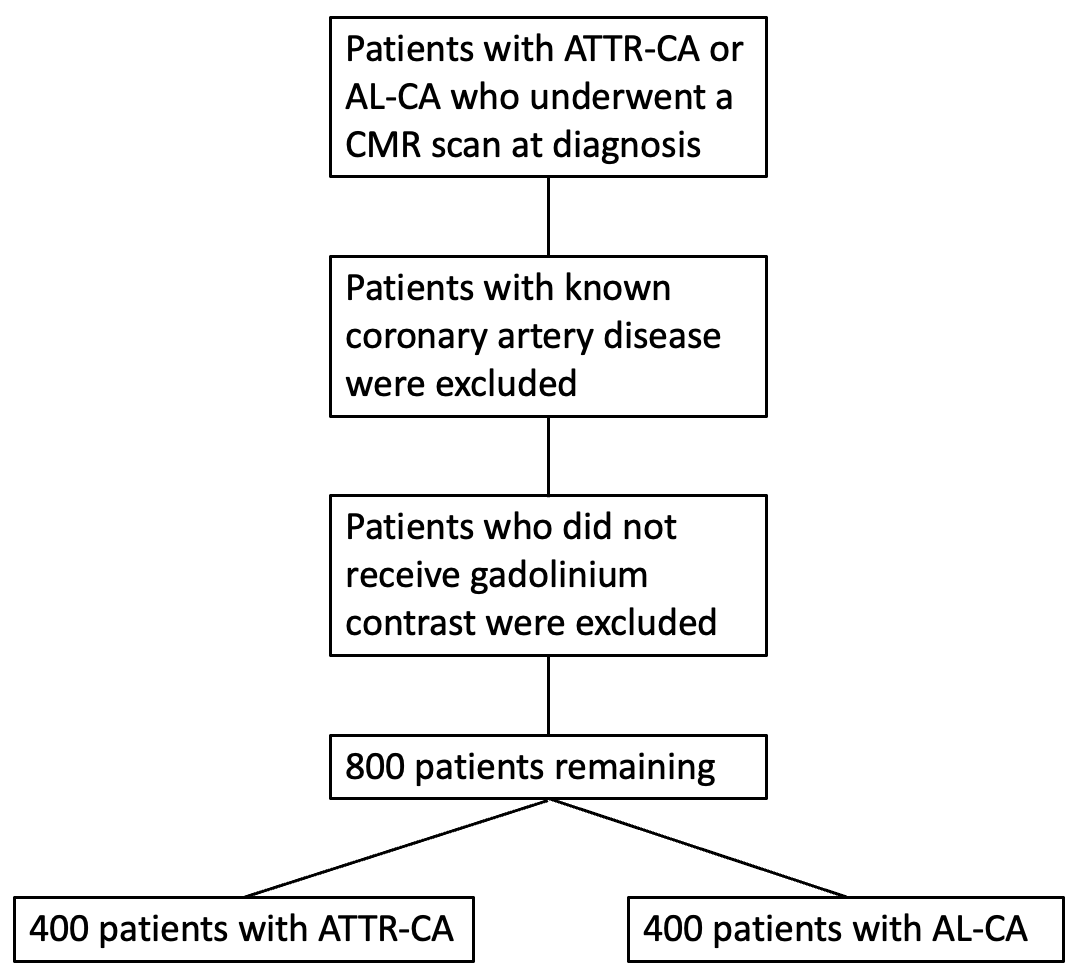


**Supplementary Figure S1.** Consort diagram detailing how patients were selected for inclusion into the study.

The number of patients excluded from the study at each stage was not documented during the data curation process and therefore is not available in this diagram.

**Supplementary Table S1**

| **Baseline characteristics of the AL-CA population stratified by absence/presence of MVO** | | | | |
| --- | --- | --- | --- | --- |
| **Baseline characteristics** | **All**  **n=400** | **No MVO**  **n=303 (76%)** | **MVO**  **n=97 (24%)** | **p value** |
| **Men** | 63% (253) | 60% (182) | 73% (71) | 0.916 |
| **Age at baseline (years)** | 66 (58 - 73) | 66 (59 - 73) | 67 (57 - 72) | 0.936 |
| **Serum biomakers** |  |  |  |  |
| **NTproBNP (ng/mL)** | 3416 (1279 - 7259) | 2703 (939 - 6180) | 5183 (2604 - 9688) | **<0.001** |
| **Troponin (ng/mL)** | 66 (36 - 130) | 59.5 (33 - 121) | 86 (47 - 148) | **0.001** |
| **eGFR (ml/min)** | 72 (55 - 90) | 73 (56 - 90) | 68 (53 - 90) | 0.269 |
| **Echocardiographic parameters** |  |  |  |  |
| **GLS (%)** | -13 (-9 ; - 17) | -14 (-10 ; -18) | -11 (-8 ; -12.6) | **<0.001** |
| **E/e’** | 15.3 (11 - 20) | 14 (11 - 20) | 16.5 (13 - 22) | **0.002** |
| **CMR parameters** |  |  |  |  |
| **LVEDV_i_ (mL/m^2^)** | 61 (51 - 71) | 60 (50 - 70) | 62 (55 - 74) | **0.014** |
| **LVESV_i_ (mL/m^2^)** | 22 (15 - 29) | 21 (14 - 28) | 26 (20 - 34) | **<0.001** |
| **MWT (mm)** | 16 (13 - 18) | 15 (13 - 17) | 17 (15 - 20) | **<0.001** |
| **LV Mass_i_ (g/m^2^)** | 98 (79 - 119) | 90 (75 - 113) | 113 (96 - 137) | **<0.001** |
| **LAA_i_ (cm^2^/m^2^)** | 14 (12 - 17) | 14 (12 - 17) | 15 (13 - 18) | **<0.001** |
| **EF (%)** | 63 (55 - 71) | 65 (57 - 73) | 58 (52 - 64) | **<0.001** |
| **SV_i_ (mL/m^2^)** | 37 (31 - 45) | 37 (31 - 46) | 36 (31 - 42) | 0.174 |
| **TAPSE (mm)** | 15 (10 - 19) | 16 (10 - 20) | 12 (9 - 16) | **<0.001** |
| **T1 (ms)** | 1177 (1136 - 1221) | 1172 (1125 - 1217) | 1188 (1158 - 1230) | **0.001** |
| **T2 (ms)** | 52 (50 - 55) | 52 (50 - 54) | 53 (50 - 56) | **0.023** |
| **Transmural LGE** | 53% (213) | 47% (143) | 72% (70) | **<0.001** |
| **RV LGE** | 71% (284) | 66% (199) | 88% (85) | **<0.001** |
| **ECV (%)** | 50 (43 - 58) | 48 (41-55) | 55 (50 - 60) | **<0.001** |

**Supplementary Table S1.** Baseline characteristics of the AL-CA population stratified by absence/presence of MVO. ECV: extracellular volume; EF: ejection fraction; eGFR: estimated glomerular filtration rate; LAA_i_: indexed-left atrial area; GLS: global longitudinal strain; LGE: late gadolinium enhancement; LVEDVi: indexed-LV end-diastolic volume; LVESVi: indexed-LV end-systolic volume; MVO: microvascular obstruction; MWT: maximal wall thickness; NTproBNP: N-terminal pro-brain natriuretic peptide; RV: right ventricle; SV_i_: indexed-stroke volume; TAPSE: tricuspid annular plane systolic excursion.

**Supplementary Table S2**

| **Baseline characteristics of the ATTR-CA population stratified by absence/presence of MVO** | | | | |
| --- | --- | --- | --- | --- |
| **Baseline characteristics** | **All**  **n=400** | **No MVO**  **n=276 (69%)** | **MVO**  **n=124 (31%)** | **p value** |
| **Men** | 89% (355) | 87% (239) | 94% (116) | **0.042** |
| **Age at baseline (years)** | 78 (73 - 82) | 78 (74 - 83) | 77 (71 - 81) | **0.035** |
| **Serum biomakers** |  |  |  |  |
| **NTproBNP (ng/mL)** | 2497 (1368 - 4976) | 2468 (1388 - 5194) | 2725 (1291 - 4876) | 0.909 |
| **Troponin (ng/mL)** | 59 (38 - 82) | 59.5 (36 - 87) | 59 (44 - 78) | 0.408 |
| **eGFR (ml/min)** | 62 (50 - 77) | 62 (50 - 76) | 62 (50 - 77) | 0.787 |
| **Echocardiographic parameters** |  |  |  |  |
| **GLS (%)** | -11 (-8 ; - 13) | -11 (-9 ; -14) | -10 (-8 ; -12.6) | **0.024** |
| **E/e’** | 16 (12 - 19) | 15 (12 – 19) | 16 (13 – 21) | 0.158 |
| **CMR parameters** |  |  |  |  |
| **LVEDV_i_ (mL/m^2^)** | 69 (59 - 81) | 68 (58 - 80) | 71 (60 - 84) | 0.383 |
| **LVESV_i_ (mL/m^2^)** | 29 (21 - 40) | 28 (21 - 40) | 32 (23 - 40) | **0.036** |
| **MWT (mm)** | 19 (17 - 22) | 19 (16 - 21) | 20 (18 - 23) | **<0.001** |
| **LV Mass_i_ (g/m^2^)** | 125 (105 - 148) | 122 (99 - 143) | 138 (115 - 159) | **<0.001** |
| **LAA_i_ (cm^2^/m^2^)** | 17 (15 - 19) | 17 (15 - 19) | 17 (15 - 20) | 0.855 |
| **EF (%)** | 57 (47 - 66) | 58 (47 - 67) | 55 (45 - 64) | 0.056 |
| **SV_i_ (mL/m^2^)** | 39 (32 - 46) | 40 (32 - 46) | 39 (30 - 45) | 0.625 |
| **TAPSE (mm)** | 11 (9 - 16) | 12 (9 - 16) | 10 (8 - 14) | **0.018** |
| **T1 (ms)** | 1142 (1116 - 1172) | 1139 (1113 - 1168) | 1150 (1121 - 1184) | **0.020** |
| **T2 (ms)** | 50 (48 - 52) | 49 (48 - 52) | 50 (48 - 52) | 0.697 |
| **Transmural LGE** | 82% (326) | 76% (210) | 94% (116) | **<0.001** |
| RV LGE | 94% (376) | 93% (257) | 96% (119) | 0.267 |
| **ECV (%)** | 55 (49 - 60) | 53 (47 - 59) | 58 (53 - 61) | **<0.001** |

**Supplementary Table S2.** Baseline characteristics of the ATTR-CA population stratified by absence/presence of MVO. ECV: extracellular volume; EF: ejection fraction; eGFR: estimated glomerular filtration rate; LAA_i_: indexed-left atrial area; GLS: global longitudinal strain; LGE: late gadolinium enhancement; LVEDVi: indexed-LV end-diastolic volume; LVESVi: indexed-LV end-systolic volume; MVO: microvascular obstruction; MWT: maximal wall thickness; NTproBNP: N-terminal pro-brain natriuretic peptide; RV: right ventricle; SV_i_: indexed-stroke volume; TAPSE: tricuspid annular plane systolic excursion.

|  | **Univariable logistic regression** | | **Multivariable logistic regression** | |
| --- | --- | --- | --- | --- |
| **Variable** | **OR (95% CI)** | **P value** | **OR (95% CI)** | **P value** |
| Age (years) | 1.00 (0.99-1.02) | 0.499 | - | - |
| Male sex | 2.06 (1.37-3.11) | 0.001 | 1.88 (1.14-3.07) | 0.012 |
| ATTR | 1.40 (1.03-1.91) | 0.033 | 0.99 (0.64-1.51) | 0.955 |
| NT-proBNP (ng/L) | 1.00 (1.00-1.00) | 0.015 | 1.00 (1.00-1.00) | 0.332 |
| Troponin-T (ng/L) | 1.00 (1.00-1.00) | 0.087 | 1.00 (1.00-1.00) | 0.915 |
| eGFR (ml/min) | 1.00 (0.99-1.00) | 0.300 | - | - |
| GLS (%) | 0.89 (0.85-0.92) | <0.001 | 0.95 (0.90-1.01) | 0.099 |
| E/e’ | 1.04 (1.02-1.07) | <0.001 | 1.01 (0.98-1.04) | 0.375 |
| LV mass indexed (g/m^2^) | 1.01 (1.00-1.01) | <0.001 | 1.00 (1.00-1.01) | 0.403 |
| Native T1 (ms) | 1.00 (1.00-1.01) | <0.001 | 1.00 (1.00-1.01) | 0.047 |
| Transmural LGE | 3.50 (2.34-5.23) | <0.001 | 1.27 (0.75-2.17) | 0.377 |
| ECV (%) | 1.08 (1.06-1.10) | <0.001 | 1.05 (1.02-1.08) | <0.001 |

**Supplementary Table S3**

**Supplementary Table S3.** Univariable and multivariable analysis of variables associated with microvascular obstruction.

| Patients with MVO: comparison between AL-CA vs ATTR-CA patients | | | | |
| --- | --- | --- | --- | --- |
| **Parameters** | **All**  **n=221** | **AL-CA**  **n=97 (44%)** | **ATTR-CA**  **n=124 (56%)** | **p value** |
| **Men** | 85 % (187) | 73 % (71) | 94 % (116) | **<0.001** |
| **Age at baseline (years)** | 72 (66.5 - 79) | 67 (57 - 72) | 77 (71 - 81) | **<0.001** |
| **Serum biomarkers** |  |  |  |  |
| **NTproBNP (ng/mL)** | 3516 (1944 - 6247) | 5184 (2602 - 9835) | 2725 (1291 - 4876) | **<0.001** |
| **Troponin (ng/mL)** | 67 (45 - 103) | 86 (47 - 148) | 59 (44 - 78) | **<0.001** |
| **eGFR (mL/min)** | 63 (51 - 79) | 67 (51 - 90) | 62 (50 - 77) | 0.052 |
| **Echocardiographic**  **parameters** |  |  |  |  |
| **GLS (%)** | -10.5 ( 7.9 ; -12.6) | -10.9 (-7.8 ; -12.6) | -10 (-8 ; -12.6) | 0.610 |
| **E/e’** | 16.4 (12.8 - 21) | 16.5 (13 - 22) | 16 (12.5 - 20.8) | 0.383 |
| **CMR parameters** |  |  |  |  |
| **LVEDVi (mL/m2)** | 66 (58 - 79) | 62 (55 - 74) | 71 (60 - 84) | **0.001** |
| **LVESVi (mL/m2)** | 29 (22 - 38) | 26 (20 - 34) | 32 (23 - 40) | **0.002** |
| **MWT (mm)** | 19 (16 - 21) | 17 (15 - 20) | 20 (18 - 23) | **<0.001** |
| **LV Massi (g/m2)** | 125 (104 - 148) | 113 (96 - 137) | 138 (115 - 159) | **<0.001** |
| **EF (%)** | 57 (48 - 64) | 58 (52 - 64) | 55 (42 - 64) | 0.052 |
| **SVi (mL/m2)** | 37 (31 - 44) | 36 (31 - 42) | 39 (30 - 45) | 0.113 |
| **LAAi (cm2/m2)** | 17 (14 - 18) | 15 (13 - 18) | 17 (15 - 20) | **<0.001** |
| **TAPSE (mm)** | 11 (8 - 15) | 12 (8 - 16) | 10 (8 - 14) | 0.266 |
| **T1 (ms)** | 1166 (1137 - 1203) | 1188 (1157 - 1230) | 1151 (1121 - 1186) | **<0.001** |
| **T2 (ms)** | 51 (49 - 54) | 53 (50 - 56) | 50 (48 - 52) | **<0.001** |
| **Transmural LGE** | 84.5% (186) | 72.2% (70) | 94.3% (116) | **<0.001** |
| **RV LGE** | 92.7% (204) | 87.6% (85) | 96.7% (119) | **<0.001** |
| **ECV (%)** | 56 (51 - 61) | 55 (50 - 60) | 58 (53 - 61) | **0.008** |

**Supplementary Table S4**

**Supplementary Table S4.** Comparison between AL-CA patients with MVO and ATTR-CA patients with MVO. AL: light chain; ATTR: Transtiretin; ECV: extracellular volume; EF: ejection fraction; eGFR: estimated glomerular filtration rate; LAAi: indexed-left atrial area; GLS: global longitudinal strain; LGE: late gadolinium enhancement; LVEDVi: indexed-LV end-diastolic volume; LVESVi: indexed-LV end-systolic volume; MVO: microvascular obstruction; MWT: maximal wall thickness; NTproBNP: N-terminal pro-brain natriuretic peptide; RV: right ventricle; SVi: indexed-stroke volume; TAPSE: tricuspid annular plane systolic excursion.
